# A large-scale investigation of alcohol-based handrub (ABHR) volume: hand coverage correlations utilizing an innovative quantitative evaluation system

**DOI:** 10.1186/s13756-021-00917-8

**Published:** 2021-03-07

**Authors:** Constantinos Voniatis, Száva Bánsághi, Andrea Ferencz, Tamás Haidegger

**Affiliations:** 1 Laboratory of Nanochemistry, Department of Biophysics and Radiation Biology, Semmelweis University, Budapest, Hungary; 2 Department of Surgical Research and Techniques, Semmelweis University, Budapest, Hungary; 3 Department of Epidemiology, Semmelweis University, Budapest, Hungary; 4 University Research and Innovation Centre (EKIK), Óbuda University, Budapest, Hungary; 5 Austrian Center for Medical Innovation and Technology (ACMIT), Wiener Neustadt, Austria

**Keywords:** Quality assurance in hand hygiene, ABHR, Hand rubbing technique, Hand coverage, ABHR volume awareness

## Abstract

**Background:**

Current hand hygiene guidelines do not provide recommendations on a specific volume for the clinical hand rubbing procedure. According to recent studies volume should be adjusted in order to achieve complete coverage. However, hand size is a parameter that highly influences the hand coverage quality when using alcohol-based handrubs (ABHR). The purpose of this study was to establish a quantitative correlation between applied ABHR volume and achieved hand coverage.

**Method:**

ABHR based hand hygiene events were evaluated utilizing a digital health device, the Semmelweis hand hygiene system with respect to coverage achieved on the skin surface. Medical students and surgical residents (N = 356) were randomly selected and given predetermined ABHR volumes. Additionally, hand sizes were calculated using specialized software developed for this purpose. Drying time, ABHR volume awareness, as well spillage awareness were documented for each hand hygiene event.

**Results:**

Hand coverage achieved during a hand hygiene event strongly depends on the applied ABHR volume. At a 1 ml dose, the uncovered hand area was approximately 7.10%, at 2 ml it decreased to 1.68%, and at 3 ml it further decreased to 1.02%. The achieved coverage is strongly correlated to hand size, nevertheless, a 3 ml applied volume proved sufficient for most hand hygiene events (84%). When applying a lower amount of ABHR (1.5 ml), even people with smaller hands failed to cover their entire hand surface. Furthermore, a 3 ml volume requires more than the guideline prescribed 20–30 s to dry. In addition, results suggest that drying time is not only affected by hand size, but perhaps other factors may be involved as well (e.g., skin temperature and degree of hydration). ABHR volumes of 3.5 ml or more were inefficient, as the disinfectant spilled while the additional rubbing time did not improve hand coverage.

**Conclusions:**

Hand sizes differ a lot among HCWs. After objectively measuring participants, the surface of the smallest hand was just over half compared to the largest hand (259 cm^2^ and 498 cm^2^, respectively). While a 3 ml ABHR volume is reasonable for medium-size hands, the need for an optimized volume of handrub for each individual is critical, as it offers several advantages. Not only it can ensure adequate hand hygiene quality, but also prevent unnecessary costs. Bluntly increasing the volume also increases spillage and therefore waste of disinfectant in the case of smaller hands. In addition, adherence could potentially decrease due to the required longer drying time, therefore, adjusting the dosage according to hand size may also increase the overall hand hygiene compliance.

## Background

Hand hygiene and hand rubbing are unequivocally the first line of defense in patient safety and even social safety in a pandemic. Since the ground-breaking discovery of Ignaz Semmelweis, hand hygiene protocols have been created, reassessed, reviewed, and rewritten [1]. In 2009 the World Health Organisation (WHO) initiated the “SAVE LIVES: Clean Your Hands” program, marking hand hygiene as the cornerstone of infection transmission prevention. Unfortunately, even today, hospital-acquired infections (HAIs) are dominantly transmitted by hands [2]. Current research is primarily focused on synthesizing more effective disinfectant agents and investigating healthcare worker compliance factors [3], however, some recent studies have raised questions about whether we are neglecting crucial factors involved in hand rubbing, and their implications on hand hygiene [4, 5]. A decade after the WHO Hand Hygiene Guideline, we now possess the technological resources [6] required to re-examine and reassess factors, which had been neglected, either due to their complexity to be measured, or were deemed insignificant and negligible.

In the worldwide-followed WHO guidelines [7, 8], the instructions regarding the application of ABHR are clear and explicit: “Apply a palmful of the product in a cupped hand, follow the 6-step protocol for 20–30 s, and cover all hand surfaces.” Initially, it may seem inconsequential, but the absence of an exact volume regarding the applied ABHR led to several issues regarding the clinical application of the guideline. The disinfectant volume does not only determine hand coverage, but also application time (drying time). To attain the desired microbial reduction on all hand surfaces, the disinfectant volume and application time are crucial.

Goroncy-Bermes et al. [9], demonstrated that an ABHR volume of 3 ml is required to obtain a sufficient microbiological reduction. Surveying the literature, it is now evident that hand size is an overlooked parameter regarding optimised hand coverage during hand hygiene [4, 10]. Zingg et al. [4], although having some limitations, concluded that for larger hands, even an ABHR volume of 3 ml might not entirely cover the entire hand surface. As written in the WHO guidelines, the term “palmful” can only be considered to be a relative form of quantification, as Healthcare Workers (HCWs) cannot objectively quantify the applied volume. At first glance, the issue seems insignificant, as logic dictates that a person with larger hands would apply more disinfectant before initiating the hand rubbing. However, a recent large-scale study [5] summarizing more than 28 million recorded hand hygiene events demonstrated how 86% of the hand rubbing events only use one push of the disinfectant applying apparatus (pump), even if this one push resulted in only 0.75 ml. Furthermore, according to Bansaghi et al. a clear decrease in volume per push is observed for numerous disinfectant dispensers [11] further indicating the presence of a disinfectant application volume issue. Variance among institutions and departments is almost certainly present, as some studies document higher volumes (3.3─3.4 ml) per hand hygiene event [12] while others lower (1.4 ml) ones [13].

Consequently, a large population of health care workers is:

- consciously not applying enough disinfectant as they are unable to reliably assess the applied volume;
- unconsciously not applying as much disinfectant as they think they are;
- not applying enough disinfectant for their particular hand size.

Hand rubbing time or application time is another focus point concerning hand rubbing quality [14]. While the WHO guidelines predicate a 20–30 s application time, some studies demonstrated that the 20–30 s application time is not enough for a 3 ml applied volume to dry on the hands [15–17], suggesting a 2 ml applied dosage. This however, may pose drawbacks as a smaller volume could potentially result in the decrease of the total disinfected hand surface [17]. In other words, for a part of the HCW population, the WHO proposed application time is only feasible when the applied volume is not sufficient to provide proper microbial reduction. Interestingly, other studies documented that an application time of 15 s is more than enough, and has no significant difference in efficacy [18–20].

Combing in the results of the aforementioned studies confirms that important open issues still exist in present hand hygiene protocols, which should be addressed and ultimately solved, as they may have serious implications, especially during a pandemic [21–23]. Proper hand hygiene is paramount to reduce coronavirus transmission and HAI rates alike [22].

Strictly speaking, a microbial reduction can only be considered sufficient once a total hand coverage is achieved, as non-disinfected areas can still transmit pathogens, or re-contaminate already disinfected hand areas. In other words, a proper microbial reduction as seen in laboratory conditions (typically on inanimate surfaces) may differ from the clinical setting. Typically, improperly disinfected or missed areas include the fingertips, dorsum of the hands and wrists [24, 25]. Unfortunately, the information found in the literature regarding infection transmission models is limited and no information was found regarding the significance of the size of non-disinfected hand areas. Nevertheless, increasing the disinfected hand area can decrease the transient flora, and therefore the infection transmission risks. While theoretically simple, defining an exact application time (for a real-life clinical setting) is rather complicated. As a HCW starts the hand rubbing and the disinfectant is being spread, the *disinfectant-volume/area ratio* (µl/cm^2^) is not constant. The ratio changes during the hand rubbing process as the ABHR is simultaneously being spread and absorbed while also evaporating. To further complicate the equation, the evaporation rate is influenced by volume, and chemical composition (e.g., alcohol concentration) [16] therefore, this dynamic relationship can only be estimated. In vitro environment application time (time required for the disinfectant to take effect and reach the standardized microbial reduction) cannot be identical with the practical in vivo application time (contact time). In practical terms, and real-life clinical scenarios, application time (time until hand rubbing results in dry hands) depends on the applied disinfectant volume and specific chemical composition of the handrub, which dominantly dictates the evaporation rate.

The primary objective of this research is the comprehensive and accurate evaluation of the ABHR *volume—coverage area* relationship. To our knowledge, no study of a similar scale exists. In addition, individual drying times, disinfectant spills and the subject’s ability to assess volume were also investigated. Ultimately, an optimized ABHR volume in addition to a proper rubbing technique would ensure total hand coverage, and consequently sufficient microbial reduction. Furthermore, this would also decrease the long-term over-application of disinfectants, which can lead to dermatological issues (e.g., skin irritation, contact dermatitis) [26, 27] for the HCWs and increased costs to the hospitals and institutions.

## Methods

**Fig. 1:**
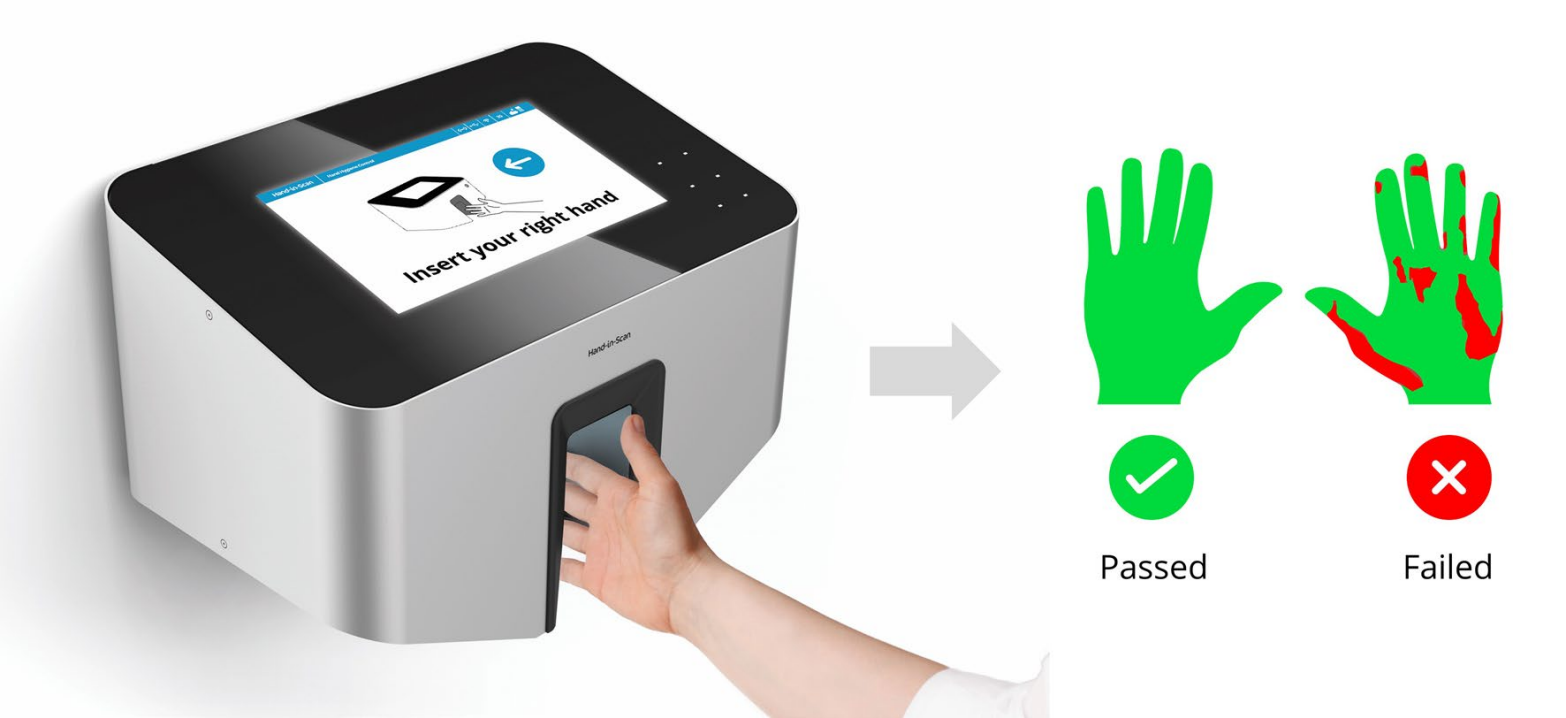
Digital health technology measurement concept employing the Semmelweis hand hygiene system, on the user interface green indicates covered areas, red indicates non-covered areas while a numeric evaluation is additionally provided (Image by HandInScan Zrt.)

A multi-site, prospective randomized study was conducted involving medical students, surgical residents and a digital tool for hand hygiene outcome evaluation. The Semmelweis hand hygiene system (HandInScan Zrt., Debrecen, Hungary) is an innovative digital health technology solution that can be utilized to visualize hand coverage after a hand hygiene event. By employing a fluorescent handrub, the device can detect the covered (and theoretically disinfected) areas down to pixel level resolution with artificial intelligence-based digital image processing [28]. The system provides an unprecedented opportunity for HCWs to directly and immediately visually observe and evaluate how effective their hand rubbing technique was (Fig. 1).

### Hand coverage measurements

The participants in this study were 3rd-year medical students (N = 298) and surgical residents (N = 57). After a brief, yet comprehensive course on hand hygiene theory, participants were assigned individual RFID (Radio Frequency Identification) cards to record their individual statistical data. Measurements involved giving participants predetermined, randomly assigned exact volumes (1, 1.5, 2, 2.5, 3, 3.5, or 4 ml). The volume range was determined based on that most professionals’ advice to use a 3 ml volume, while surveys found that in practice, smaller amounts were commonly used. An investigator using a Dispensette S Analog-adjustable bottle-top dispenser (Brand GmbH, Germany) gave the participants the exact (but undisclosed) volume to the centre of their dominant hand’s palm. After performing the hand hygiene event using a fluorescent dye containing ABHR solution, participants' hands were assessed using the Semmelweis System. Measurements were performed under the direct supervision of an investigator. All measurements were performed with the Semmelweis Training Rub (HandInScan Zrt.) a liquid ABHR containing 70% ethanol and a regulatory-wise insignificant amount (i.e., < 0.02%) of fluorescent dye. Medical student hand hygiene performance was evaluated every second week (up to 5 occasions) while residents were evaluated on a daily basis (up to 10 occasions).

### Drying time measurements

Drying time (start of hand rubbing until completely dried hands) was measured using a stopwatch. Participants were given a signal to initiate the WHO 6-step hand rubbing protocol. As soon as participants felt that their hands are dry, they indicated to the investigator, who recorded and documented the elapsed time in seconds.

### Hand size determination

Hand size calculation is essential, as the applied ABHR *volume/area* quotient would be different among different populations. After initial calibration, hand sizes were calculated digitally by measuring the pixels of the scanned images [29]. Improperly recorded images were filtered and excluded from the calculations. Good quality images were used for hand size (hand surface area in cm^2^) determination according to an already established Automated Area Assessment Method determined automatically by the Semmelweis System. In some cases, participants were asked to draw around the outline of their non-dominant hand on a sheet of paper. Subsequently, the drawings were used and hand sizes were also determined by a Digital Outline Assessment Method as a control. The results using the two methods were compared to examine the precision of the Semmelweis System method.

### ABHR spill and volume awareness

Both the medical students and the surgical residents were given two questions to answer directly before the evaluation with the Semmelweis System. Important to note that all measurements were performed as blinded studies. Neither the medical students, nor the surgical residents were informed about the exact volume they received during the measurements.

### Statistical analysis

Where applicable, a statistical analysis of the results was performed. According to the predictor and outcome variables, different statistical tests were chosen. For the statistical analysis, R Core Team: R: A Language and Environment for Statistical Computing (R Foundation for Statistical Computing, Vienna, Austria R Version: 4 0.0 Released: 2020. 04.24) was used. To concurrently investigate the effect of the person’s experience (student or resident), handrub volume, hand size and covered area, a linear mixed-effect model was fitted on the data. The logarithm of the missed area (%) as the outcome variable was explained by the handrub amount (ml), the hand size (cm^2^), the participant’s experience (student or resident) and the interaction effect of handrub volume and hand size (specific hand coverage). The random intercept was the individual error for participants. Compound symmetry correlation structure for handrub amount and different power variance structure for handrub at different participants was used to fit the final model. When examining how handrub volume and hand size affect drying time, a generalized least square regression model was used. Finally, when investigating possible correlation between volume awareness (Table 1 Question 2) and hand coverage, a chi square test was performed. The questionnaire’s answers (predictor values) and the coverage results (expected values) were regarded as binary parameters (Not enough = 0, Just right, Too much = 1, while Coverage < 95% = 0, Coverage > 95% = 1). Throughout the analysis, results were designated as significant when *p* < 0.05.

**Table 1:** Hand hygiene event questionnaire completed by all participants

Question	Possible answers
Did the disinfectant spill from your hand during the rubbing?	Yes/No/I don’t know
How did you find the disinfectant volume?	Not enough/Just right/Too much

## Results

### Disinfectant coverage results

**Fig. 2:**
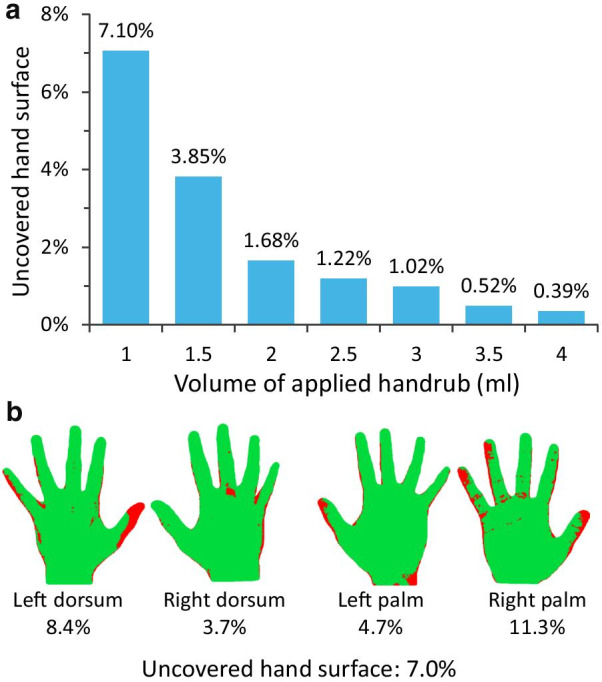
**a** Hand coverage (percent of the total hand surface missed during hand hygiene event). **b** Example of the computed outcome: missed areas at 7% uncovered surface

The assessment methodology proved to be simple and straightforward. The majority of the participants readily understood the concept, and adhered to the measuring parameters. After the hand assessment events, 50 measurements were filtered and removed due to faulty measurements or software-generated artefacts. All events are plotted in Fig. 2, where a direct correlation between disinfectant volume and hand coverage can be observed as increasing the volume results in smaller missed areas. The final number of examined hand hygiene events was 1622. The non-covered areas fell to less than 1% only with a dose of 3.5 ml or higher. Interestingly, not everyone covered their hands perfectly (100%) even with a 4 ml dose. Noteworthy to mention that when comparing the standardized volumes of 1.5 and 3 ml, it is apparent that half the volume equals less than half of the coverage, thus volume is not linearly proportional to coverage.

**Fig. 3:**
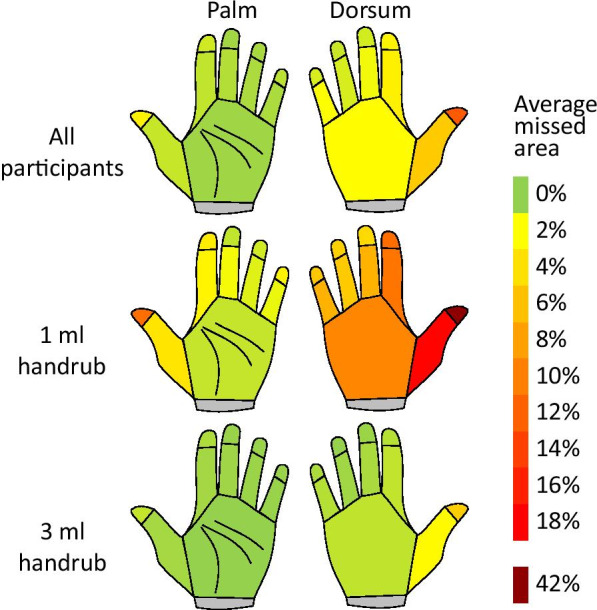
Typically missed areas during the hand hygiene events. (ALL—N = 1557, 1.5 ml—N = 153, 3 ml—N = 724)

Missed areas vary according to the applied ABHR volume. Typically missed areas included the fingertips, the thumb and the dorsum of the hand. The correlation of volumes and missed areas is exhibited in Fig. 3.

### Hand size determination

**Fig. 4:**
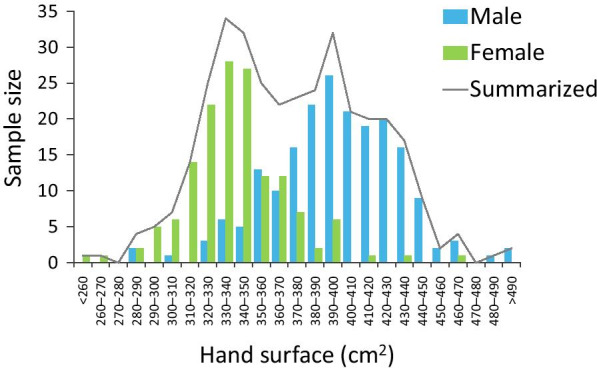
Hand size calculation results, provided by the Semmelweis System’s software (automated area assessment method)

**Fig. 5:**
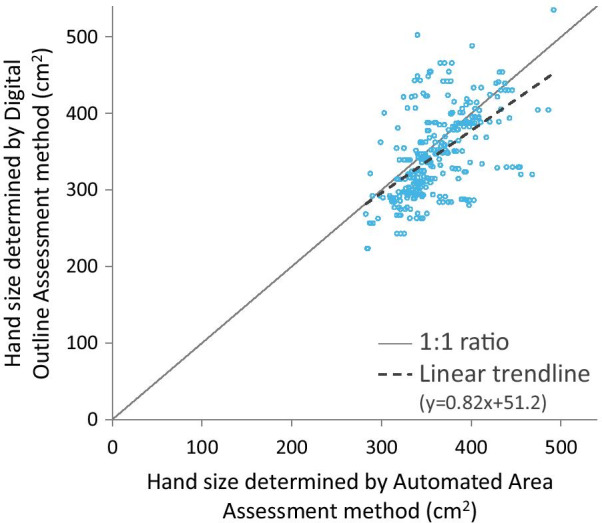
Comparing the two different hand size determination methods. Note: Average hand size difference between the two methods was 10.1%

Digital hand size assessment results can be observed in Fig. 4. To be as objective as possible for the disinfectant coverage measurements, hand size was calculated by the Automated Area Assessment (AAA) method for every single hand hygiene event. Individual hand size values (from the same participant) were then averaged. Results exhibit a bimodal distribution. Comparing the currently utilized (Automated Area Assessment) method with a manually determined method (Digital Outline Assessment) results are almost identical (differences measuring 10%) (Fig. 5) confirming therefore that software provided machine-based automated hand size calculations can be considered reliable. Interesting to note that the differences between the two methods were documented when larger hands were assessed. Inaccuracies in the digital outline assessment method, most likely resulted by the experimental settings, i.e., participants were asked to draw around their hands, and not all participants put the same effort resulting in significantly lower precision.

### Specific hand coverage (μl/cm^2^)

**Fig. 6:**
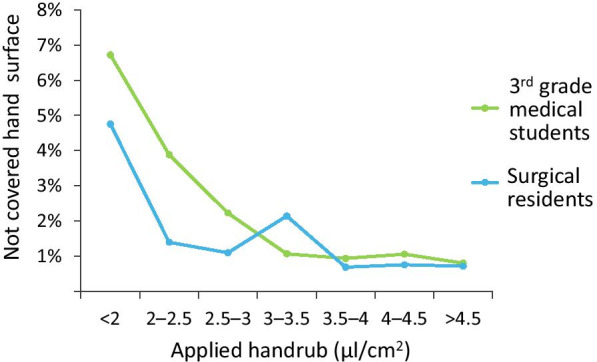
Specific Hand Coverage in the case of 3rd grade medical students, who recently learned proper hand disinfection technique, and surgical residents, who have several years of experience. Applying enough ABHR, medical students reached similar results as surgical residents. When suboptimal volumes were used experience had indeed an impact

In order to objectively assess the effect of ABHR volume on hand coverage, specific hand coverage was determined to calculate the *disinfectant volume per hand surface area* ratio. According to our results, the average total hand size area was 372.9 cm^2^ while the median hand size was 370.2 cm^2^. When 3 ml of handrub is distributed on a pair of on average-sized hands (372.9 cm^2^ each) it equals a 4.02 μl/cm^2^ specific hand coverage. In Fig. 6, it can be seen that surgical residents performed considerably better than 3rd-year medical students. Thus, if a 4.02 μl/cm^2^ is regarded as sufficient for an average hand, the 3 ml volume seems to be a valid approximation as to what a medium hand-sized person should apply.

**Fig. 7:**
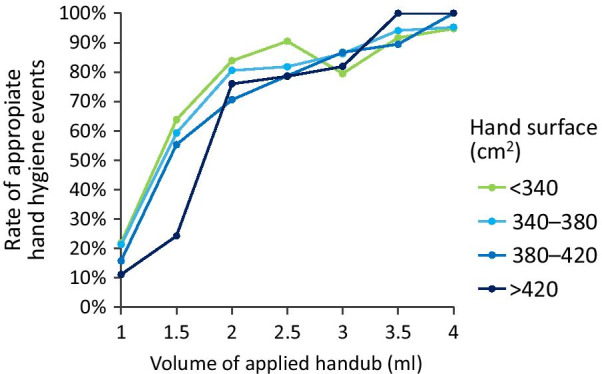
Hand size (hand surface)—uncovered area correlation measurements

Regarding hand size and applied disinfectant coverage, Fig. 7 visually demonstrates how at a smaller dose the difference between covered areas due to hand sizes becomes evident. As the volume of the applied disinfectant increases, the gap between the hand sizes decreases.

### Drying time

**Fig. 8:**
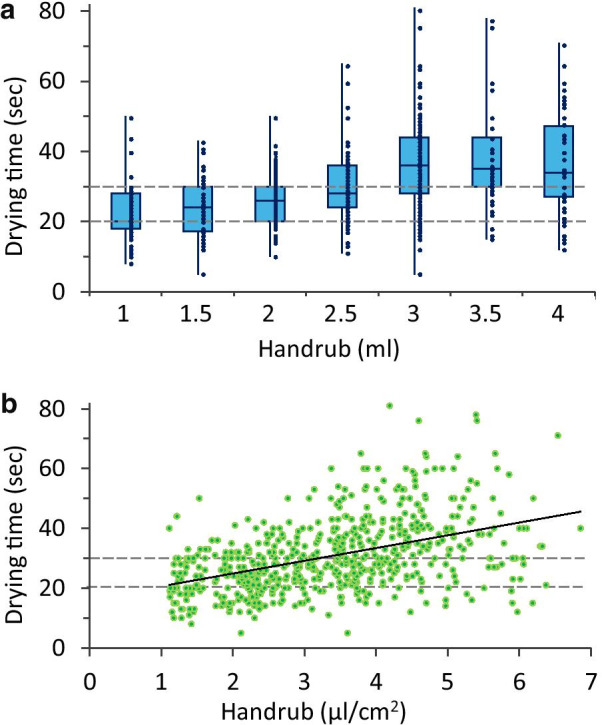
Results of drying time measurements. **a** Drying time and volume correlation without taking hand size into consideration **b** Drying time and Specific Hand Coverage correlation (hand size taken into consideration). Note: WHO guidelines mention that hand rubbing should take 20–30 s, while the typically prescribed 2.5–3 ml ABHR volume requires longer time to dry

Drying time results confirm that by increasing the disinfectant volume, the application time (drying time) increases as well. At a 3 ml volume, a plateau is reached in the mean values (Fig. 8). Important to note that these results concern an ABHR with a 70% ethanol concentration, differences, may be observed using other products. Furthermore, the resulted relatively wide variances indicate that (apart from hand size) other intrinsic or extrinsic factors can potentially influence drying times as well.

### Disinfectant spill and volume awareness

**Fig. 9:**
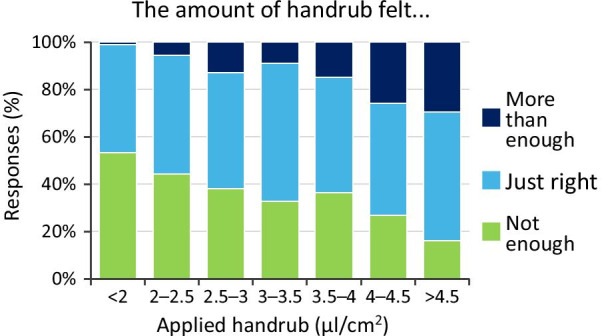
Volume awareness results (Volume awareness refers to the ability of the participants to assess the ABHR volume given to them). Note: 1.5 ml and 3 ml ABHR volumes on an average sized hand result in 4.02 and 2.51 μl/cm^2^

**Fig. 10:**
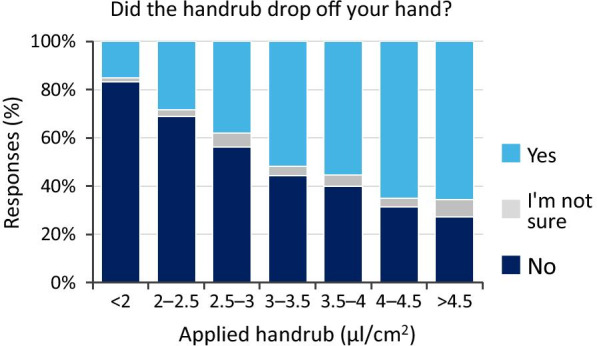
Handrub spillage results (Handrub spillage refers to the ABHR spillage during a hand hygiene event). Note: 1.5 ml and 3 ml ABHR volumes on an average sized hand resulted in 2.51 and 4.01 μl/cm^2^, respectively

The increase in the applied ABHR volume (while being undisclosed) was generally consciously detected among the participants. However, it was clearly shown that participants cannot precisely assess the given volumes, as even with a 3 ml dose (on average-sized hands resulting in a 4.02 µl/cm^2^ specific hand coverage) a significant number of students (more than 30%) (Fig. 9) felt that the given ABHR volume was not enough. It is also evident that increasing the volume of the applied disinfectant increases the hand rubbing volume losses during the WHO 6-step protocol (Fig. 10). Interestingly, at the same *disinfectant-volume/area* ratio values the participants felt that the given volume was not enough and yet the disinfectant was dripping from their hands. A further remark is that investigators reported, that although some participants did not report dripping, some spill was still observed. Arguably, dripping can occur without the participants noticing it therefore, the actual results are probably an underestimation compared to the questionnaire.

### Statistical analysis results

According to the data analysis, the handrub volume (*p* < 0.0001), hand size (*p* < 0.0001), and participant’s experience (medical student or surgical resident) (*p* = 0.003) have a significant effect on the non-covered area. In addition, the disinfectant volume/hand size ratio also has a significant effect on the size of the missed area (*p* = 0.0339). Regarding drying times disinfectant volume is a significant factor (*p* < 0.0001), while hand size is not (*p* = 0.083). Finally, participant volume awareness seems to correlate with achieving the predetermined (> 95%) hand coverage (*p* = 0.034).

## Discussion

This is the first objective, digital health technology supported, large-scale investigation regarding disinfectant volume and hand coverage correlations. Unlike several other studies where the observer or examiner calculated or evaluated the disinfectant-covered areas manually, during our investigation evaluations were all performed using an objective computerised and automated electronic system [6].

Comparing the performance of medical students and surgical residents it is clear that experience and procedural memory are key to perfect the hand rubbing technique and hand hygiene protocol (Fig. 5). Nevertheless, a perfect score (0% missed areas) was rarely achieved. An important condition was that measurements occurred always under the direct supervision of an investigator. Thus, the overall performance is significantly (yet uniformly) influenced by the Hawthorne effect. In a real-world clinical setting, the performance should be worse not only due to less attention being given to the WHO 6-step protocol but due to the often decreased applied disinfectant volume as well [11].

Regarding disinfectant volume and hand coverage correlations, our results indicate that a volume of 1.5 ml is inadequate as it leaves on average 4% of the total hand surface bare of disinfectant. As Goroncy-Bermes et al. [9] suggested, a volume of 3 ml seems rather appropriate as according to our findings the missed area falls under 1%. While increasing the disinfectant dose to 3, 3.5 and 4 ml decreases the missed area to approximately 1%, 0.7% and 0.5%, respectively, and the average drying time for these volumes was well above the recommended 20─30 s according to the current WHO guideline, which may put unnecessary load on the HCWs during clinical practice. Our results concur with the results of Macinga et al. and Suchomel et al. [14, 16] who also documented increased drying times. A drying time of 20 s was uncommon even in the cases of 1 ml applied volumes. Contradictions in the literature exist as Paula et al. findings [19] demonstrate how hand wettability is statistically equal after a 15 or 30 s application time. However, as they stated their results may have been obscured due to the small population they examined (N = 20), the effect of the continuous training, or that hand size was not used to normalize results among participants. Further investigating the results, it can be seen that drying times even when made independent from hand size demonstrate a large variance. This suggests that other intrinsic factors are influencing the results (e.g., skin temperature, skin hydration).

Furthermore, application time and microbiological reduction should be re-examined as controversy still exists in the current research. For example, Pires et al. or Harnoss et al. [18] investigated the application time and bacterial reduction relationship. Their results suggest that no significant difference was found between 15 and 30 s application time. Their studies included a rather limited and probably experienced study population (N = 32 and 14 respectively), whilst no hand size measurements were taken into consideration, and only the microbiological sampling on fingertips was performed.

When incorporating hand size in the evaluations, our results demonstrate how hand size does matter. 1.5 ml of disinfectant is clearly not sufficient for larger hands. At 3 ml volume, smaller and larger handed participants achieved almost the same percentage of disinfected areas.

As results show, 3 ml is a good approximation for medium size hands yet an optimized volume for each individual is seemingly the best option [10] as just plainly increasing the volume also increases spillage and therefore a waste of disinfectant in the case of smaller hands.

Apart from the material (and therefore financial) waste, the findings of Greenaway et al. and others [18, 30] suggest that compliance also decreases with higher applied volumes (due to longer application time). According to our results, applied ABHR volumes are relative to the same volume for small-handed people the volume feels excessive while for large-handed ones feel just right. Therefore, adjusting the dosage according to hand size could also increase the overall compliance with the protocol. In addition to an optimized volume, simplifying the hand hygiene steps could also decrease the spillage while also increasing compliance [31].

### Limitation of the study

Due to the natural distribution among a population, larger and smaller handed people are fewer than medium-size handed people therefore the number of recorded measurements per hand size is not truly equal. Therefore, the volume of the disinfectant spill (while probably different for the two populations) was not quantified and was additionally overshadowed by the average values. Consequently, the actually applied volume (actual volume = given volume − spillage) was different for the two hand sized populations. In addition, the effect of alcohol type and concentration was not investigated in this study, only a 70% ethanol containing handrub was tested. Handrubs with different alcohol concentrations can have different characteristics. Finally, medical students and surgical residents participated in the study thus intermediate experience level was not investigated.

## Conclusions

According to our results, the covered area during a hand hygiene event strongly depends on the applied ABHR volume. At small volumes (i.e., 1–1.5 ml), the covered area deficit is more evident, as people with larger hands fail to cover the entire hand surface. A 3 ml applied volume is sufficient for medium size hands to achieve full coverage, however, this volume requires more than the instructed 20–30 s to be thoroughly applied. In addition, this volume can be insufficient for larger hands, but wasteful for smaller ones as not only the disinfectant loss (spillage) will increase. Notably, the additional rubbing (drying) time is disadvantageous, since hand coverage will not increase. The optimal applied ABHR volume is therefore relative. The implementation of an optimised, clinical set, hand size depended protocol would benefit future hand hygiene guidelines, as it would not only increase the speed and efficiency of hand hygiene events, but also improve compliance and adherence rates while keeping the disinfectant wastes and costs to a minimum.

## Data Availability

The datasets used and/or analysed during the current study are available from the corresponding author on reasonable request.

## Abbreviations

ABHR: alcohol-based handrub
HCW: healthcare workers
WHO: World Health Organization
